# Promotion of self-directed learning abilities among Chinese medical students through preparing for career calling and enhancing teaching competencies in medical education: a cross-sectional study

**DOI:** 10.1186/s12909-024-05330-4

**Published:** 2024-04-08

**Authors:** Chen-xi Zhao, Zi-jiao Wang, Xiao-jing Yang, Xing Ma, Ying Cui, Yan-xin Zhang, Xin-hui Cheng, Shu-e Zhang, Qing-feng Guo, De-pin Cao

**Affiliations:** 1 Academic Affairs Office, First Affiliated Hospital of Harbin Medical University, 150001 Harbin, China; 2 Department of Health Management, School of Health Management, Harbin Medical University, 150081 Harbin, China; 3 Center for the Evaluation of Higher Education Teaching and Learning of Harbin Medical University, 150081 Harbin, China

**Keywords:** Self-directed learning ability, Career calling, Teaching competencies, Medical students, Scale development, Moderate effect, †*

## Abstract

**Background:**

Medical students face a heavy burden as they are tasked with acquiring a vast amount of medical knowledge within a limited time frame. Self-directed learning (SDL) has become crucial for efficient and ongoing learning among medical students. However, effective ways to foster SDL ability among Chinese medical students are lacking, and limited studies have identified factors that impact the SDL ability of medical students. This makes it challenging for educators to develop targeted strategies to improve students’ SDL ability. This study aims to assess SDL ability among Chinese medical students and examine the effects of career calling and teaching competencies on SDL ability, as well as the possible mechanisms linking them.

**Methods:**

Data were collected from 3614 respondents (effective response rate = 60.11%) using cross-sectional online questionnaires and analyzed using IBM SPSS Statistics 22.0. The questionnaire comprised a Demographic Characteristics Questionnaire, Self-directed Learning Ability Scale (Cronbach’s alpha = 0.962), Teaching Competencies Scale, and Career Calling Scale.

**Results:**

The average SDL ability score of Chinese medical students was 3.68 ± 0.56, indicating a moderate level of SDL ability. The six factors of the Self-directed Learning Ability Scale—self-reflection, ability to use learning methods, ability to set study plans, ability to set studying objectives, ability to adjust psychological state, and willpower in studying—accounted for 12.90%, 12.89%, 12.39%, 11.94%, 11.34%, and 8.67% of the variance, respectively. Furthermore, career calling was positively associated with SDL learning ability (*β* = 0.295, *p* < 0.001), and SDL learning ability was positively associated with teaching competencies (*β* = 0.191, *p* < 0.01). Simple slope analysis showed that when the level of teaching competencies was higher, the influence of career calling on SDL ability was stronger.

**Conclusions:**

Chinese medical students’ SDL ability has room for improvement. Medical students could strengthen their willpower in studying by setting milestones goals with rewards, which could inspire their motivation for the next goals. Teachers should guide students to learn experience to improve students’ reflective ability. Educators play a crucial role in bridging the gap between career calling education and SDL ability enhancement, highlighting the significance of optimal teaching competencies. Colleges should focus on strengthening teachers’ sense of career calling and teaching competencies.

## Background

Medical education functions in a highly dynamic environment [1]. Both during their time in college and in their future professional careers, medical students need to continually update their medical knowledge to maintain their clinical skills [2]. They are required to acquire more information than students of other subjects and expected to develop a lifelong learning ability [3]. However, the overwhelming volume of medical information [4], extensive literature [5], and limited time [6] impose a huge burden on medical students. In the face of these challenges and the evolving nature of medical education, it has become an urgent challenge for educators to help develop efficient and ongoing self-directed learning (SDL) abilities among medical students.

After Malcolm Knowles first introduced the concept of SDL, several scholars understood that SDL would significantly advance medical students’ careers [5–8]. Since then, educators have increasingly focused on medical students’ abilities to effectively plan their time and acquire the necessary professional skills for their future careers. Holec deeply studied the concept of SDL ability, considering it the capability to take responsibility for one’s learning [9], including setting learning objectives, deciding on learning content and pace, choosing learning methods and technologies, monitoring learning progress, and evaluating learning outcomes. SDL has gradually become an indispensable competency for medical students [10], helping them stay abreast of the latest medical advancements [11]. Without a sense of SDL for academic learning, medical students may struggle to meet professional demands in the present and future [12]. Therefore, it is critical to integrate a sense of SDL into daily life to adequately prepare medical students for their careers.

Nevertheless, many medical students struggle with effective time management [13]. Due to the traditional exam-oriented education system in China, some Chinese medical students, accustomed to acquiring knowledge from teachers, remain passive learners with limited learning activity [14, 15]. These students may not have the ability to learn independently when faced with problems [16] and may lack a sense of learning consciousness [17], ultimately lagging behind the pace of medical development. This is a significant problem that could affect the quality of medical services in China in the coming years. To become outstanding medical practitioners, Chinese medical students must urgently enhance their SDL ability. Fostering an adaptive and sustainable SDL ability among Chinese medical students is an imminent requirement that medical educators must address.

Before proposing appropriate strategies to enhance this ability, it is imperative to evaluate the current SDL ability of Chinese medical students. However, after reviewing the literature, we found minimal research on measuring the current level of SDL ability among medical students. Therefore, we required a suitable tool to assess the current SDL ability of Chinese medical students and identify effective strategies to enhance it. Based on previous literature, this study explored two factors that may have a significant influence on Chinese medical students: career calling [18, 19], and teaching competencies [20, 21].

In recent years, there has been an increasing focus on medical practitioners’ career calling. Scholars have defined career calling as a subjective experience in which individuals are determined to work voluntarily and positively [22], indicating a passion or drive toward working in a particular field [23]. Bunderson noted that when individuals strongly identify with their jobs, they tend to focus all their attention on work [24]. Moreover, a high level of career calling is related to positive emotions [25], and this active feeling can lead to proactive behaviors [26]. In essence, career calling can maintain the passion of medical students for learning, encouraging them to actively plan and conduct their studies [27]. Can it be extrapolated that the higher the career calling of a Chinese medical student, the better their SDL ability? Based on these predictions, career calling may serve as a protective factor in fostering SDL ability. Therefore, this study aimed to explore the relationship between career calling and SDL ability, along with constructive factors to mobilize SDL ability.

Teaching philosophy reflects an individual’s beliefs and values about teaching and learning. It discusses the self-identity of teachers and how they educate others [28]. Thus, teachers can play the role of a bridge between career calling education and medical students’ learning. Teachers with strong beliefs and values related to career calling may influence students in a subtle way. Thus, it is important to focus on teachers’ teaching capacity. Studies have defined teaching competencies as comprising teachers’ personal characteristics, knowledge, skills, and attitudes required in various teaching environments [29]. Teachers with appropriate characteristics that align with educational requirements can benefit students’ academic achievements [30]. Deep subject knowledge can also improve students’ grades [31], while good teaching skills can direct students’ focus toward learning [32]. Additionally, positive attitudes toward teaching can promote students’ positive attitudes [33]. Overall, teachers’ teaching competencies influence students’ learning and academic achievements and are highly significant for nurturing future talents. Therefore, this study posits that teaching competencies play a positive moderating role between Chinese medical students’ career calling and SDL ability. In practical teaching, teachers’ teaching competencies directly impact students, who observe and judge these competencies more objectively and comprehensively. To better evaluate teaching competencies, this study used “students’ perception of their teachers’ competencies in teaching” as an evaluation method.

This study aimed to measure Chinese students’ SDL ability level and explore the correlations among career calling, teaching competencies, and SDL ability. To accomplish these aims, we proposed the following two hypotheses:

### Hypothesis 1

Career calling is positively associated with SDL ability among Chinese medical students.

### Hypothesis 2

Teaching competencies positively moderate the relationship between career calling and SDL ability among Chinese medical students.

## Methods

### Ethics statement

The procedures of this study adhered to the guidelines of the Declaration of Helsinki and were reviewed and approved by the Ethics Committee of the Institutional Review Board of Harbin Medical University(ECHMU: HMU202072). Each participant provided written online informed consent before participating in this study. All data collected from the participants were kept anonymous and confidential to protect their privacy.

### Survey design and data collection

Initially, according to the calculation method and standard requirements for the cross-sectional sample size based on Zhou et al. [34], the minimum sample size for this study was calculated to be 1824. Considering a minimum response rate of approximately 40% based on previous online survey experience, the sample size was expanded to 4560. To further ensure data quality, we determined the final number of respondents to be 6000.

After determining the sample size, six medical universities were selected based on their size, academic programs, research performance, admission scores, and number of students. Different specialties and grades were then randomly selected in each university. These universities are located in Nanjing, Guangzhou, Dalian, Harbin, Mudanjiang, and Daqing.

To ensure the cost-effectiveness, time-effectiveness, and accessibility of the study [35], a cross-sectional anonymous online survey was conducted using a multistage stratified convenient sampling method to collect data from medical students from July to September 2021. Based on the characteristics of medical students, we used a multi-staged stratified convenient sampling method, with quotas allocated by the division of students’ years and majors. First, we grouped medical students according to their majors. Next, we further divided these groups into smaller groups based on their years. Finally, we distributed questionnaires and received responses in accordance with the predetermined quantity. The survey was conducted through the online survey platform “Questionnaire Star.” The researchers monitored the collected questionnaires in real-time through the platform and used it to effectively manage the data.

### Data quality control

Data quality is key to ensuring the reliability and validity of a study. In this study, a data quality control process was implemented in three stages: questionnaire design, survey administration, and data processing.

### Questionnaire design

The questionnaire included three “seriousness test questions” placed at the beginning, middle, and end. These questions prompted respondents to select specific answers to test their seriousness [36]. Additionally, a “self-evaluation question of answer quality” was included at the end of the questionnaire for respondents to evaluate the quality of the questionnaire. Each participant was allowed to respond only once.

### Survey administration

One or two research leaders were selected from each university to conduct an “accurate survey” of the target participants. This ensured that all the questionnaires were completed by the target groups.

### Data processing

During data processing, strict data screening criteria were applied. Responses with incorrect selections to any of the “seriousness test questions” were deleted. Respondents who took less than three hundred seconds to complete the questionnaire were considered “speeders,” and their questionnaires were deleted. Questionnaires that participants suggested deleting were also excluded. Finally, each remaining questionnaire was reviewed by the authors, and those with an irregular distribution of answers were deleted.

### Study instruments

A Demographic Characteristics Questionnaire, Self-directed Learning Ability Scale, Teaching Competencies Scale, and Career Calling Scale were used. Permissions were obtained for using the Teaching Competencies Scale and the Career Calling Scale.

### Measurement of demographic characteristics

Eight demographic information was collected using a self-designed questionnaire: gender, grade, major, experience of leadership, hometown, monthly living expenses, parenting style, and education level of parents. Student grade was collected as a continuous variable ranging from 1 to 5. The majors of students were categorized into eight groups: “basic medical science,” “clinical medicine,” “stomatology,” “public health,” “pharmacy,” “medical technology,” “nursing,” and “others.” Leadership experience was divided into “student leaders” and “ordinary students.” Students’ hometowns were categorized as “rural” or “urban.” The monthly living expenses of students were categorized into four groups: RMB “0 ∼ 1000”, “1000 ∼ 1500”, “1500 ∼ 2000,” and “2000 and above”. Parenting style was divided into four categories: “neglecting,” “permissive,” “authoritarian,” and “authoritative.” The education level of parents was categorized as “primary school or below,” “junior middle school,” “high school,” “junior college,” or “bachelor’s degree or above.”

### Measurement of SDL ability

According to the definition of SDL ability in previous studies [37, 38], SDL ability was divided into six dimensions: ability to set studying objectives, willpower in studying, ability to set study plans, ability to use learning methods, ability to adjust psychological state, and ability to self-reflect. A 28-item instrument designed by the authors was used to measure SDL ability level. In a previously published article, the self-designed SDL ability scale was tested and implemented [39]. To ensure the applicability of the scale, a pre-survey was conducted with 454 students, which showed good reliability and validity. Items were scored on a 5-point Likert scale ranging from 1 “totally inconsistent” to 5 “totally consistent,” with higher scores representing a higher degree of SDL ability.

### Measurement of teaching competencies

The teaching competencies of the teachers were assessed using a 5-item Teaching Competencies Scale, a questionnaire developed for German students by Thomas and Müller [40]. Items were scored on a 5-point Likert scale ranging from 1 “totally not in line with” to 5 “fully in line with,” with higher scores indicating a higher level of teachers’ teaching competencies. The cross-cultural adaptation of the scale into Chinese included performing forward and backward translations, with an assessment of its cultural equivalence and clarity. High reliability was demonstrated in the reliability analysis, with a Cronbach’s α-coefficient of 0.943 for the scale in this study.

### Measurement of career calling

Medical students’ career calling level was assessed using the 4-item Career Calling Scale revised by Dik et al. [41]. The scale has been cross-culturally adapted and verified in other studies in China [42, 43]. Items were scored on a 5-point Likert scale ranging from 1 “never” to 5 “every day,” with higher scores indicating a higher degree of career calling. Cronbach’s α-coefficient for the Career Calling Scale in this study was 0.843.

### Statistical analysis

This study utilized Amos version 24.0 software and SPSS version 22.0 for statistical analysis, and a two-tailed *p* < 0.05 was considered statistically significant. We assessed the suitability of the data for factor analysis by conducting the Kaiser–Meyer–Olkin (KMO) measure of sampling adequacy and Bartlett’s *χ* [2] test of sphericity. Subsequently, exploratory factor analysis (EFA) was conducted to explore the causal structure. We employed the principal component analysis (PCA) and varimax-rotation method to extract six factors, removing items with factor loadings lower than 0.4.

Confirmatory factor analysis (CFA) with maximum likelihood was used to validate the factor structure of the Self-directed Learning Ability Scale. Various indexes were used to assess model fit, including root mean square error of approximation (RMSEA), goodness-of-fit index (GFI), adjusted goodness-of-fit index (AGFI), and normed fit index (NFI), among others. The normed chi-square (*χ* [2]) and goodness-of-fit test (*χ*^*2*^*/df*) were used to evaluate the null hypothesis that the model fits the data. However, achieving this in large sample sizes can be challenging, so we utilized the aforementioned indexes as criteria for our analysis. Cronbach’s alpha coefficient was used to measure the reliability of our instrument.

We used descriptive statistics and frequencies to analyze the demographic variables and total scores of the three scales. SDL ability scores across different demographic categories were examined using independent samples *t*-test or one-way ANOVA. In cases where one-way ANOVAs were found to be significant, we conducted least-significant-difference (LSD) tests for multiple comparisons. Pearson correlation analysis was used to examine correlations among SDL ability, teaching competencies, and career calling. Hierarchical multiple regression analysis was employed to test the moderating effect of teaching competencies on the relationship between career calling and SDL ability. All variables related to SDL ability in univariate analysis (*p* < 0.05) were included in the hierarchical multiple regression model. We performed the model estimation using PROCESS, a convenient, free, and easy-to-use computational add-on for SPSS documented by Hayes [44]. Before conducting the regression analysis for moderating effects, we employed mean centering (subtracting raw scores from the mean) to mitigate multicollinearity.

## Results

### Results of EFA, CFA, and reliability

A total of 6012 students were invited to participate in this study. Of these, 3614 questionnaires were completed and passed the quality control procedure, yielding a response rate of 60.11%. Results from both KMO and Bartlett’s tests demonstrated that the samples met the criteria for factor analysis criteria, with a KMO measure of sampling adequacy of 0.975.

The six-factor model explained 70.12% of the variance, with each factor contributing as follows: ability to self-reflect (12.90%), ability to use learning methods (12.89%), ability to set study plans (12.39%), ability to set studying objectives (11.94%), ability to adjust psychological state (11.34%), and willpower in studying (8.67%). The pattern and structures of the rotated common factors are shown in Table 1.

**Table 1:** Rotated factor loading matrix of all items

Items	Factor Loading
Factor 1: Ability to set studying objectives	
A1. I can determine my learning objectives by myself.	0.745
A2. I have short-term learning objectives (daily or weekly).	0.738
A3. I have long-term learning goals (semester or academic year).	0.729
A4. After completing a learning objective, I will determine the next learning objective as soon as possible.	0.733
Factor 2: Willpower in studying	
B1. Even if the content of the final exam is a lot, I can stay up late and finish the review.	0.723
B2. When there is a conflict between learning and entertainment, I will not affect learning due to entertainment.	0.587
B3. In learning, I can do an important but boring thing for a long time.	0.623
B4. No matter what difficulties I encounter, I will stick to my learning goal.	0.552
B5. I think I have strong self-discipline in the process of learning.	0.552
Factor 3: Ability to set studying plans	
C1. I can arrange my study time reasonably.	0.613
C2. I can break down a rough learning goal into multi-stage learning steps.	0.604
C3. I know exactly what I should learn every day.	0.623
C4. I can make plans in my heart on how to complete the learning tasks of each week.	0.612
C5. Before I start learning, I ask myself, “what should I learn next?” And other related issues.	0.543
Factor 4: Ability to use learning methods	
D1. I take the initiative to learn some efficient learning methods.	0.681
D2. I can use appropriate learning methods according to different learning contents.	0.644
D3. I keep improving my learning methods.	0.683
D4. I can use many ways to solve the problems encountered in learning (such as asking teachers, searching on the Internet, etc.).	0.673
D5. I pay great attention to observing and learning from other people’s learning methods and experiences.	0.659
Factor 5: Ability to adjust psychological state	
E1. I am always full of energy to learn.	0.487
E2. I have ways to prevent the bad emotions that arise during study.	0.794
E3. I have ways to ease the bad emotions that arise during study.	0.838
E4. When anxiety occurs in my learning process, I will think of some relaxed and happy things to get rid of anxiety.	0.806
Factor 6: Ability to Self-reflect	
F1. I can summarize a stage of learning.	0.632
F2. Before I go to bed, I often think about what I learned today and how well did I learn.	0.732
F3. After each stage of study, I would think about whether I had completed the study plan.	0.683
F4. I evaluate my learning effect regularly.	0.722
F5. I regularly reflect on how to improve my learning.	0.671

**Fig. 1:**
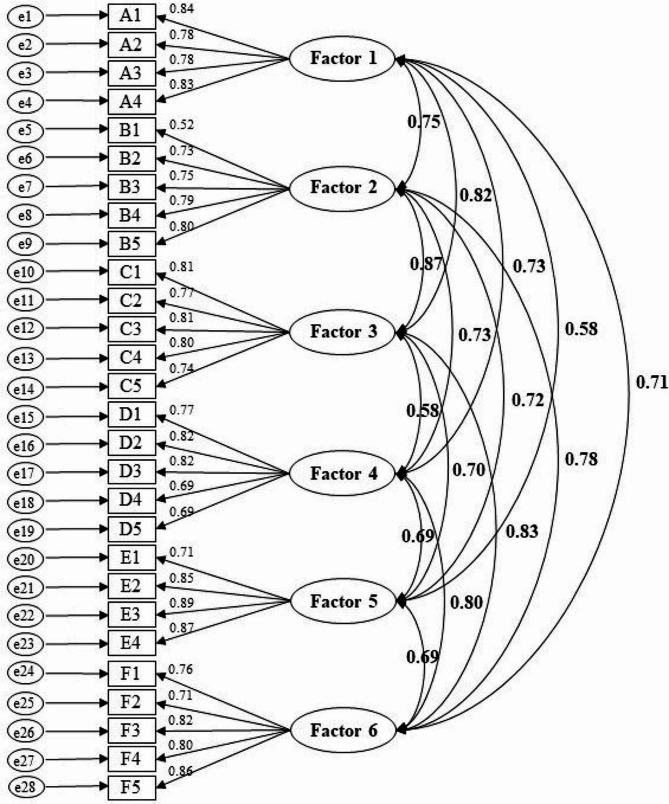
Path diagram for the model with standardized parameter estimates

The results of the CFA are presented in Table 2. The model fit the data reasonably well, with GFI, AGFI, NFI, and RMSEA all indicating a good fit. While the *χ*^*2*^*/df* was slightly higher than ideal, it is considered acceptable in large sample sizes [45]. The path diagram with standardized parameter estimates is shown in Fig. 1.

**Table 2:** Summary of fit indices

Indexes	χ^2^/df	GFI	AGFI	NFI	RMSEA (90% CI)
Coefficients	8.50	0.946	0.934	0.959	0.046

In this study, Cronbach’s alpha coefficients were used to assess the internal reliability of the instrument. The Cronbach’s alpha coefficient for the total score was 0.962. The alphas for the sub-scales of ability to set studying objectives, willpower in studying, ability to set study plans, ability to use learning methods, ability to adjust psychological state, and ability to self-reflect were 0.88, 0.84, 0.89, 0.87, 0.90, and 0.89, respectively.

### Current SDL ability level among Chinese medical students

**Fig. 2:**
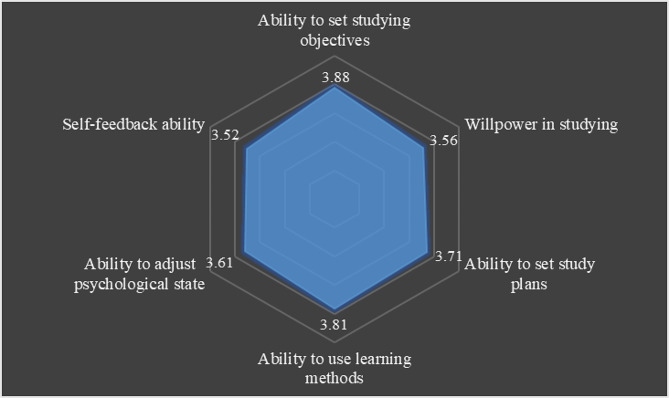
Radar chart of SDL ability among chinese medical students

The results indicated that the SDL ability among Chinese medical students was at a moderate level (*M* = 3.68, *SD* = 0.56). The scores for specific aspects of SDL ability, from highest to lowest, included the ability to set studying objectives (*M* = 3.88, *SD* = 0.68), ability to use learning methods (*M* = 3.81, *SD* = 0.61), ability to set study plans (*M* = 3.71, *SD* = 0.65), ability to adjust psychological state (*M* = 3.61, *SD* = 0.70), willpower in studying (*M* = 3.56, *SD* = 0.65), and ability to self-reflect (*M* = 3.52, *SD* = 0.70), as presented in Table 3; Fig. 2.

**Table 3:** The Means (*M*), Standard Deviations (*SD*) score of SDL ability among Chinese medical students (*n* = 3,614)

Dimension	M ± SD	Min-Max	Rank
Ability to set studying objectives	3.88 ± 0.68	1–5	1
Willpower in studying	3.56 ± 0.65	1–5	5
Ability to set study plans	3.71 ± 0.65	1–5	3
Ability to use learning methods	3.81 ± 0.61	1–5	2
Ability to adjust psychological state	3.61 ± 0.70	1–5	4
Ability to self-reflect	3.52 ± 0.70	1–5	6
Self-directed learning ability	3.68 ± 0.56	1–5	−

### Difference in SDL ability based on participant characteristics

Significant differences were observed in SDL ability scores depending on students’ demographics, including gender, grade, major, experience of leadership, hometown, monthly living expenses, parenting style, and education level of parents. Details of the scores under different demographic characteristics and LSD test results are detailed in Table 4. The demographic breakdown of participants indicated that 74.41% were women and 25.59% were men. Regarding grades, the majority were freshmen (56.79%), followed by sophomores (18.26%). In terms of majors, 4.10% were in basic medicine, 34.26% in clinical medicine, 6.34% in stomatology, 4.73% in public health and preventive medicine, 15.55% in pharmacy, 11.73% in medical technology, 14.78% in nursing, and 8.52% in other majors. Over half of the participants (53.90%) had experience as student leaders. Regarding hometowns, 55.76% were registered as urban residents, while the rest were from rural areas. In addition, 45.63% of students reported monthly living expenses between 1000 ∼ 1500 RMB. Regarding parenting types, the majority of students (61.23%) reported experiencing permissive parenting. Finally, the education levels of the participants’ parents varied as follows: primary school or below (5.67%), junior middle school (32.57%), high school (28.17%), junior college (12.04%), and bachelor’s degree or above (21.56%).

**Table 4:** Sample characteristics and one-way ANOVA analysis / independent samples *t*-test of SDL ability of Chinese medical students

Characteristics	n	%	M ± SD	F/t	*P*	LSD
Gender						
①Male	925	25.59	3.73 ± 0.61	2.805	0.005^*^	①>②
②Female	2689	74.41	3.66 ± 0.54			
Grade						
①Freshman	2085	57.69	3.73 ± 0.55	11.769	0.000^**^	①>② ①>③ ①>④
②Sophomore	660	18.26	3.60 ± 0.57			
③Junior	608	16.82	3.64 ± 0.56			
④Senior and above	261	7.22	3.61 ± 0.59			
Major						
①Basic medical science	148	4.10	3.72 ± 0.52	9.618	0.000^**^	①>⑦, ②>③ ②>⑦, ②>⑤ ③>⑦, ②>⑥ ④>⑦, ②>⑦ ⑤>⑦, ②>⑧ ⑥>⑦, ⑧>⑦
②Clinical Medicine	1238	34.26	3.76 ± 0.57			
③Stomatology	229	6.34	3.64 ± 0.61			
④Public health	171	4.73	3.73 ± 0.59			
⑤Pharmacy	562	15.55	3.70 ± 0.55			
⑥Medical technology	424	11.73	3.64 ± 0.55			
⑦Nursing	534	14.78	3.53 ± 0.56			
⑧Others	308	8.52	3.64 ± 0.51			
Experience of leaders						
①Student leaders	1948	53.90	3.74 ± 0.57	6.997	0.000^*^	①>②
②Ordinary students	1666	46.10	3.61 ± 0.55			
Hometown						
①Rural	1599	44.24	3.63 ± 0.53	5.042	0.000^*^	②>①
②Urban	2015	55.76	3.72 ± 0.59			
Monthly living expenses						
①(≤0, 1000) RMB	302	8.36	3.71 ± 0.56	3.751	0.011^**^	④>②
②(1000–1500) RMB	1649	45.63	3.65 ± 0.55			
③(1500–2000) RMB	1149	31.79	3.69 ± 0.57			
④(2000, ∞) RMB	514	14.22	3.74 ± 0.61			
Parenting style						
①Neglecting parenting	312	8.63	3.43 ± 0.58	30.237	0.000^**^	④>②>① ④>③>①
②Permissive parenting	2213	61.23	3.68 ± 0.56			
③Authoritarian parenting	362	10.02	3.67 ± 0.52			
④Authoritative parenting	727	20.12	3.79 ± 0.55			
Education degree of parents						
①Primary school or below	205	5.67	3.57 ± 0.49	9.262	0.000^**^	⑤>③>① ⑤>④>① ⑤>④>②
②Junior middle school	1177	32.57	3.64 ± 0.56			
③High school	1018	28.17	3.67 ± 0.55			
④Junior college	435	12.04	3.70 ± 0.55			
⑤Bachelor’s degree or above	779	21.56	3.77 ± 0.60			

Note: ^*^ Independent Samples *t*-Test ^**^ One-Way ANOVA

### Correlations among continuous variables

Table 5 presents the correlations among SDL ability, teaching competencies, and career calling. The three variables were found to be significantly correlated with each other. The level of SDL ability was positively correlated with career calling and teaching competencies. Career calling was positively correlated with teaching competencies. Therefore, Hypothesis 1 was supported.

**Table 5:** Means, Standard Deviation (*SD*) and correlations of continuous variables (*n* = 3614)

Variables	Range	M ± SD	1	2	3
1. Self-directed learning ability	1–5	3.68 ± 0.56	1		
2. Career calling	1–5	3.72 ± 0.70	0.439^**^	1	
3. Teaching competencies	1–5	4.08 ± 0.63	0.485^**^	0.335^**^	1

Note: ^**^P < 0.01; the Pearson Correlation is significant at the 0.01 level (two-tailed)

### Career calling, Teaching competencies, and SDL ability

**Fig. 3:**
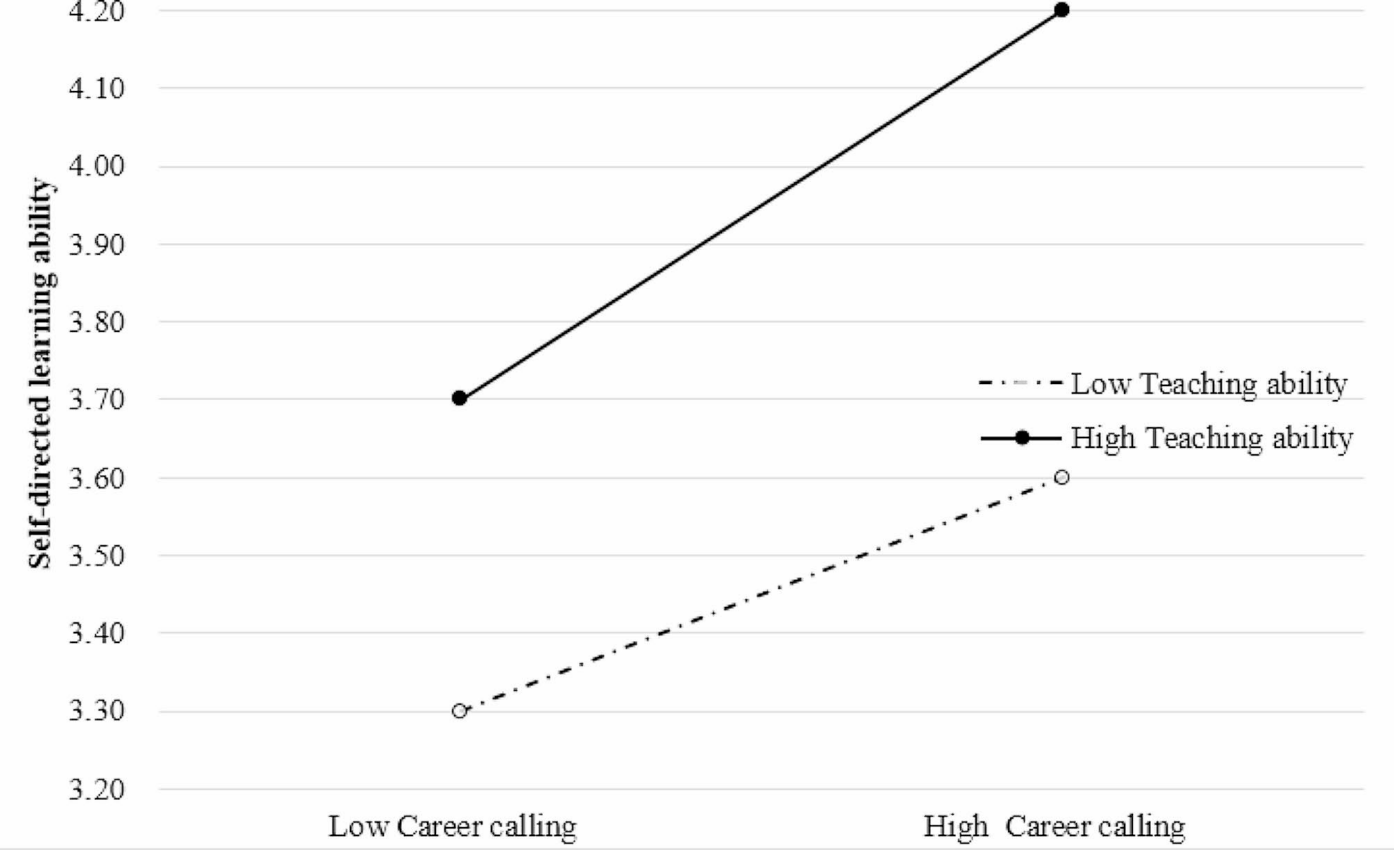
Moderated effect of teaching competencies on the association between career calling and SDL ability

Following the suggestions by Aiken and West [46], the data were centered (by subtracting the average value), indicating that teaching competencies significantly moderated the association between career calling and SDL ability, as shown in Table 6; Fig. 3. Therefore, Hypothesis 2 was confirmed, suggesting that teaching competencies positively moderated the relationship between career calling and SDL ability among Chinese medical students.

**Table 6:** Hierarchical multiple regression model (*n* = 3,614)

Variables	Self-directed learning ability			
	M1(β)	M2(β)	M3(β)	
Control variables				
Gender	-0.027	-0.022	-0.022	
Grade	-0.083^***^	0.003	0.003	
Major	-0.091^***^	-0.053^***^	-0.053^***^	
Experience of leaders	-0.108^***^	-0.072^***^	-0.071^***^	
Hometown	0.042^*^	0.034^*^	0.032	
Monthly living expenses	-0.027	-0.022	-0.022	
Parenting style	0.106^***^	0.054^***^	0.054^***^	
Education degree of parents	0.032	0.027	0.028	
Moderator				
Career calling		0.295^***^	0.068	
Independent variable				
Teaching competencies		0.375^***^	0.191^**^	
Interaction				
Teaching competencies × Career calling			0.340^**^	
*F*	23.547^***^	183.862^***^	168.365^***^	
*R* ^2^	0.05^***^	0.338^***^	0.340^***^	
*ΔR* ^2^	0.05^***^	0.288^***^	0.290^***^	

Note: ^*^P < 0.05 ^**^P < 0.01 ^***^P < 0.001

## Discussion

### SDL ability among Chinese medical students

This study investigated the level of SDL ability in Chinese medical students. The results derived from this study indicate that the instrument measuring SDL ability had high reliability and validity, with Cronbach’s α exceeding 0.90 and the designed six-factor structure confirmed by CFA. The standardized factor loading coefficient of the items and the cumulative variance contribution rate also confirmed the reliability and validity of the instrument. In summary, the instrument appears to be appropriate for the assessment of SDL ability among Chinese medical students.

The mean score of SDL ability among surveyed medical students was 3.68 ± 0.56 *(Mean* ± *SD*). Similar results were found by Yang et al. [47], suggesting that the SDL ability of Chinese medical students is at a moderate level. Among demographic characteristics, gender, grade, major, experience of leadership, hometown, monthly living expenses, parenting style, and education level of parents were found to have an impact on the SDL ability of Chinese medical students.

The results of the scoring order of SDL ability in each dimension indicate that Chinese medical students can actively set studying objectives and plans, and are able to use learning methods correctly. However, in the process of SDL, the ability to adjust psychological state, willpower in studying, and ability to self-reflect were relatively low. This indicates that students’ executive ability and reflective ability were poor, affecting the effectiveness of SDL, or even making the study plan formalistic. Scholars have found that students may be disturbed by minor distractions before fully engaging in the learning process, leading to potential disruptions in their ability to execute their learning goals, even when they have meticulously planned their studying routines in advance [48]. Medical students could add milestones achievement rewards to their study plans. The sense of achievement gained from completing small milestones of learning goals could inspire medical students to move on to the next goal, and thus enhance their willpower to learn. Additionally, studies have pointed out that students may face challenges in describing the influence without drawing lessons from experience [49]; such reflection may be ineffective. In this context, teachers could guide medical students to delve deeply into their experiences hidden behind various events, thereby improving their reflective ability.

### Career calling and its positive association with SDL ability among Chinese medical students

The findings of this study confirm that career calling can positively affect SDL ability among Chinese medical students. This result is similar to Lang’s findings, which suggest that students with a strong career calling or a steadfast commitment to their professions tend to have higher levels of energy and a greater sense of control over their professional success [50]. For medical students, a stronger sense of career calling is associated with a greater SDL ability. Chinese medical students’ societal value is closely tied to their academic skills. Additionally, the medical industry requires its workers to cultivate the capacity for lifelong learning to keep up with the latest developments [51]. In essence, Chinese medical students must continue learning on the job to maintain their societal value and status. Therefore, Chinese medical students are encouraged to develop SDL abilities during their undergraduate education to become qualified medical practitioners and smoothly transition into formal work.

It is therefore crucial for medical universities to devote sufficient effort to developing medical students’ sense of career calling during higher education. Chinese medical universities could implement a series of curriculum changes focused on the missions of the medical profession, aiming to enhance students’ sense of responsibility and morality, which in turn would promote self-regulation in learning. By using real-life cases to highlight the responsibilities that medical practitioners bear concerning human life, Chinese medical universities can help students cultivate a noble sense of career calling. This can motivate students to invest more energy and time in academic learning, leading to higher academic achievements through enhanced SDL abilities.

### Moderating role of teaching competencies in the positive association between career calling and SDL ability among Chinese medical students

This study provides evidence that teaching competencies can play a positive moderating role between Chinese medical students’ career calling and SDL ability. Strong teaching competencies can capture students’ attention during lectures. For instance, medical teachers can use engaging teaching techniques to make the transfer of seemingly dull knowledge interesting and memorable. Teaching competencies, such as a passionate teaching attitude, can inspire students to unlock their learning potential [52]. Accordingly, when students’ learning potential is unleashed, teachers’ strong teaching competencies, coupled with a broad knowledge base, can cater to students’ academic curiosities. This mutual relationship can stimulate students’ interest, leading them to immerse themselves in learning and create a virtuous cycle. As a result, Chinese medical students may recognize the significance of SDL and proactively improve their SDL ability.

The path to a medical professional learning career is undoubtedly challenging and lengthy, but it should not lack academic assistance and motivation. Properly combining extrinsic teaching competencies with intrinsic career calling can provide the physical and psychological energy needed for medical students to advance further. Therefore, from the perspective of medical colleges, addressing how to improve medical teachers’ teaching competencies seems to be an urgent issue. Medical colleges could design questionnaires to evaluate existing teaching competencies and gather feedback from students to target improvements in teachers’ teaching competencies. Moreover, colleges could invite teachers from other disciplines to deliver lectures and provide training for medical teachers, as few medical teachers have received systematic educational training and may lack knowledge in educational theory and practice [53]. Enhancing teaching competencies is an ongoing endeavor, but it can greatly assist Chinese medical students in improving their SDL abilities and optimizing the quality of education.

### Limitations

Although the present study reveals important findings, it has some limitations. First, the data collected are cross-sectional, indicating that establishing causal relationships among the factors was not possible. Second, considering that each medical college has its own unique circumstances and our sample did not include all medical students in China, the generalizability of the results may be limited. Third, the questionnaires were collected online, which may have introduced response bias and made it challenging to control data quality. In future studies, scholars could consider researching a wider and more diverse sample through face-to-face investigations to address these limitations.

## Conclusions

This study displayed the research and development process of the SDL ability and verified the reliability and validity of the SDL ability scale for 6 factor 28 items once again. We found that Chinese medical students’ SDL ability is at a moderate level, suggesting room for improvement. We also identified eight demographic factors that influence Chinese medical students’ SDL ability and explored the relationships among career calling, teaching competencies, and SDL ability. Both career calling and teaching competencies were found to be effective factors that can strengthen Chinese medical students’ SDL ability.

## Data Availability

The datasets used and/or analyzed during this study are available from the corresponding authors on reasonable request hydzhangshue@163.com.

## Abbreviations

SDL: Self-directed learning
KMO: Kaiser–Meyer–Olkin
EFA: Exploratory factor analysis
PCA: Principal component analysis
CFA: Confirmatory factor analysis
RMSEA: Root mean square error of approximation
GFI: Goodness-of-fit index
AGFI: Adjusted goodness-of-fit index
NFI: Normed fit index
LSD: Least-significant-difference
